# Use of Transcutaneous Auricular Vagus Nerve Stimulation as an Adjuvant Therapy for the Depressive Symptoms of COVID-19: A Literature Review

**DOI:** 10.3389/fpsyt.2021.765106

**Published:** 2021-12-15

**Authors:** Zhi-Peng Guo, Peter Sörös, Zhu-Qing Zhang, Ming-Hao Yang, Dan Liao, Chun-Hong Liu

**Affiliations:** ^1^Beijing Hospital of Traditional Chinese Medicine, Capital Medical University, Beijing, China; ^2^Research Center Neurosensory Science, Carl von Ossietzky University of Oldenburg, Oldenburg, Germany; ^3^Beijing Key Laboratory of Acupuncture Neuromodulation, Beijing Institute of Traditional Chinese Medicine, Beijing, China

**Keywords:** transcutaneous auricular vagus nerve stimulation, COVID-19, brain circuits, depression, epidemic

## Abstract

The coronavirus disease 2019 (COVID-19) comprises more than just severe acute respiratory syndrome. It also interacts with the cardiovascular, nervous, renal, and immune systems at multiple levels, increasing morbidity in patients with underlying cardiometabolic conditions and inducing myocardial injury or dysfunction. Transcutaneous auricular vagus nerve stimulation (taVNS), which is derived from auricular acupuncture, has become a popular therapy that is increasingly accessible to the general public in modern China. Here, we begin by outlining the historical background of taVNS, and then describe important links between dysfunction in proinflammatory cytokine release and related multiorgan damage in COVID-19. Furthermore, we emphasize the important relationships between proinflammatory cytokines and depressive symptoms. Finally, we discuss how taVNS improves immune function via the cholinergic anti-inflammatory pathway and modulates brain circuits via the hypothalamic–pituitary–adrenal axis, making taVNS an important treatment for depressive symptoms on post-COVID-19 sequelae. Our review suggests that the link between anti-inflammatory processes and brain circuits could be a potential target for treating COVID-19-related multiorgan damage, as well as depressive symptoms using taVNS.

## Background

In December 2019, a novel coronavirus disease (COVID-19) outbreak emerged from Wuhan, Hubei Province, China, initiating a global health threat and posing a challenge to the psychological resilience of populations worldwide (1). Clinically, presentation of COVID-19 varies from being asymptomatic, to including mild symptoms such as fever, sore throat, headache, fatigue, to manifesting as severe acute respiratory distress syndrome (ARDS) (2). Moreover, it also interacts with the cardiovascular, nervous, renal, and immune systems at multiple levels (3). An extreme immune reaction resulting in elevated levels of inflammatory cytokines, often referred to as a cytokine storm, has been linked to an increased number of deaths from COVID-19 (4, 5). However, even worse than this, the COVID-19 pandemic has also led to an increased prevalence of mental health problems, such as difficulty sleeping, depression and anxiety, and hypomania (6). Although a number of vaccines have been proved to be effective (7, 8), evidence-based evaluations and interventions targeting mental health disorders are relatively scarce (9). Transcutaneous auricular vagus nerve stimulation (taVNS) is being explored as an adjuvant therapy to the depressive symptoms of COVID-19 during the pandemic to deal with these disorders.

The concept of taVNS as a therapy has emerged relatively recently. The technique makes use of the analgesic effects of the neuronal network that innervates the vagus nerve (10), which targets the cutaneous receptive field of the auricular branch of the vagus nerve at the outer ear (11). Promising results indicate that, following taVNS treatment, the symptoms of mood disorders can be alleviated painlessly and without the need for surgery (12). Ventureyra was the first to propose applying vagus nerve stimulation (VNS) using surgically implanted electrodes wrapped around the vagus nerve in the neck (10). In 2005, VNS was approved as a long-term adjunctive treatment for patients with refractory depression of more than 18 years of age (13, 14). From a neuroanatomical point of view, vagus nerve fibers project to the nucleus tractus solitarius (NTS) and the locus coeruleus (LC), where they form direct and indirect ascending projections to many brain regions, including the midbrain, hypothalamus, amygdala, hippocampus, and frontal lobe (15). The vagus nerve, which is the longest nerve in the body, connects the central nervous system to the body by innervating major visceral organs such as the liver, spleen, and gastrointestinal tract (16). Once an inflammatory response has been detected, taVNS may help to attenuate inflammatory responses via the cholinergic anti-inflammatory pathway and by modulating brain circuits via the hypothalamic–pituitary–adrenal (HPA) axis (3, 17). Acute respiratory distress syndrome (ARDS) or fulminant pneumonia can lead to widespread inflammation and very high concentrations of cytokines in the lungs, accompanied by activation of the anti-inflammatory pathways mentioned above (18). To date, clinical and laboratory research demonstrated that taVNS can improve lung function (19, 20). In addition, taVNS is commonly used to treat encephalopathy, encephalitis, ischemic infarcts, cerebral venous thrombosis, as well as peripheral nervous system pathologies [i.e., muscle injuries, and peripheral neuropathies; (21–26)].

In order to better understand the mechanisms underlying taVNS, we review the literature on proinflammatory cytokines and the brain imaging correlates of taVNS. To date, there have not been any reviews that considered in detail how taVNS might treat depressive symptoms, which develops from COVID-19, or its associated co-morbidities. We provide an integrated account of how the dysregulation of inflammatory and immunological responses affect brain circuits in COVID-19.

## Historical Background of taVNS

**Figure 1:**
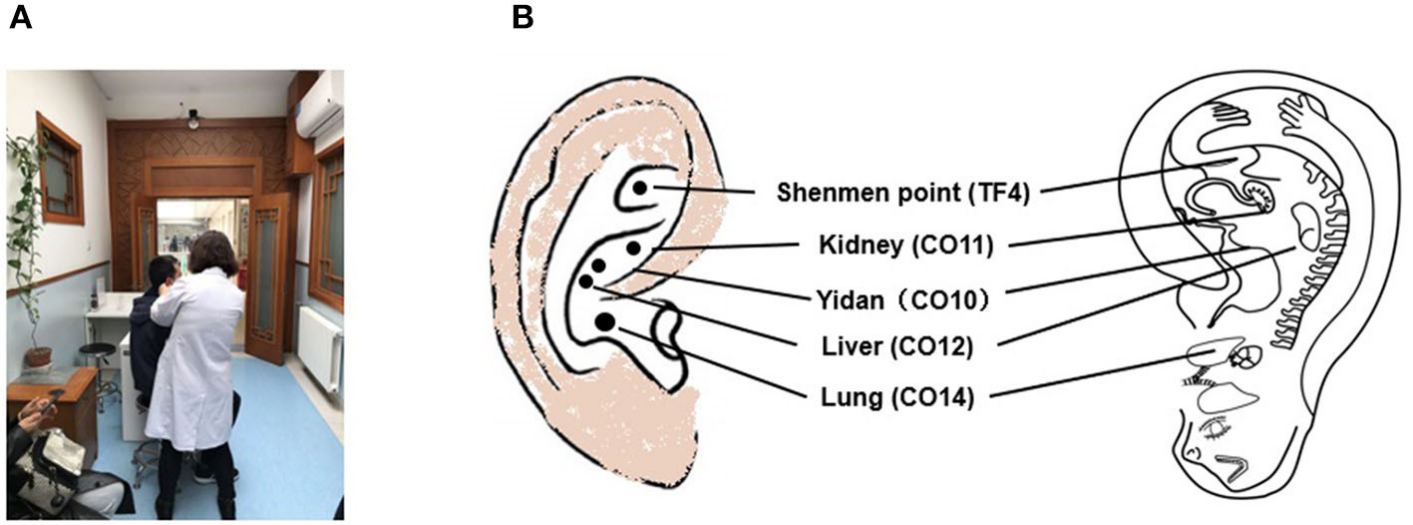
**(A)** Auricular acupuncture practice has recently been attracting the attention of the public in China and is commonly carried out within the Chinese medical hospital system. **(B)** TF4 and CO10-12 are used to stimulate the auricular branch of the vagus nerve, with the outer ear corresponding to an “inverted fetus map” [according to King and Hickey, 2013 (27)].

Auricular acupuncture originated in China during the Chou period (first millennium BCE) and has recently attracted scientific and public attention as it becomes increasingly accessible to the general public in modern China (Figure 1A) (28). The practice of auricular acupuncture is referenced in the Huangdi Neijing (The Yellow Emperor's Classics of Internal Medicine), which describes how the ear is not isolated but rather is directly or indirectly connected with 12 meridians (six yang and six yin) (29). In the 1950s, Dr. Paul Nogier, a French neurologist, proposed that the outer ear represents “an inverted fetus map” (Figure 1B) (30)]. In 1990, the World Health Organization (WHO) recognized auricular acupuncture as a self-contained microacupuncture system that maps all portions of the ear to specific parts of the body and to the internal organs (31). Having considered the anatomy of the neural pathways in the external auricle, Usichenko et al. proposed that the analgesic effects of auricular acupuncture could be explained by stimulation of the auricular branch of the vagus nerve (32). The vagus is known to be a mixed nerve, with about 80% of its fibers carrying sensory afferent information to the brain and about 20% carrying efferent motor information to the liver, spleen, and gastrointestinal tract (33). Thus, it is very likely that taVNS functions based on the Chinese system of energy circulation along the meridians, which connect “diseased” body organs with the external auricle. In addition to Asian countries, in which this technique is widely available and easy to apply, it may be possible to use taVNS to effectively respond to the COVID-19 pandemic-related depressive symptoms as well as multiorgan damage in environments where medical resources are limited.

## The Important Link Between Proinflammatory Cytokines and Covid-19 Pandemic-Related Multiorgan Damage

Several studies have suggested that the pathogenesis of COVID-19 involves an inability to resolve the inflammatory response along with the activation of immune cells and inflammatory cytokines (18, 34). In COVID-19 patients, an unregulated inflammatory response to the infection can result in the dysregulation of T cells with associated lymphopenia, high levels of the proinflammatory cytokines interleukin (IL)-6 and tumor necrosis factor (TNF)-α, and high levels of inflammatory chemokines, including C-C motif chemokine ligand (CCL-2) (35). In a study by Staats et al., 49-year-old man with excessive fatigue, mental cloudiness and body aches, and mental cloudiness had ceased after 5 days non-invasive VNS therapy (19). Furthermore, the authors also summarized five studies that used taVNS to treat COVID-19 and reported that the majority of patients obtain relief from respiratory distress after taVNS therapy. Three review papers have hypothesized that the cytokine storm and the worsening of patient health can be ameliorated or even prevented by taVNS (3, 18, 36). Therefore, targeting the inflammatory response and immune cells using taVNS might be a promising line of research in the fight against COVID-19-related inflammatory cytokine-induced multiorgan damage.

Current research indicates that COVID-19 might involve multiple organs including those in the central and peripheral nervous systems, rather than being restricted to the respiratory system (37). Recently, it has been noted that COVID-19 patients experience a number of different neurological symptoms, such as headache, dizziness, hyposmia, and hypogeusia during the course of the illness (38). Psychiatric symptoms, including post-traumatic stress disorder (PTSD), anxiety, and depressive symptoms, have also been reported in patients with COVID-19 (39, 40). Even worse than this, Kremer et al. found signal abnormalities in the medial temporal lobe and non-confluent multifocal white matter hyperintense lesions (41). Post-mortem brain imaging has demonstrated subcortical hemorrhagic and cortico-subcortical edematous changes, as well as olfactory impairment in patients who died of COVID-19 (42). Based on the results of published studies, COVID-19 encephalopathy appears to be more common in cases comorbid for encephalopathy, encephalitis, acute disseminated encephalomyelitis, myelitis, meningitis, ischemic infarcts, or cerebral venous thrombosis (43). In the peripheral nervous system, COVID-19 has been associated with dysfunction in the sense of smell and taste, and with muscle injury (41). Of note, the etiology of the encephalopathy in COVID-19 mentioned above is mostly linked to injury of the central and peripheral nervous systems by a cytokine storm, blood clots, or direct damage to specific receptors (41, 44). The pathogen that causes COVID-19, severe acute respiratory syndrome coronavirus 2, can invade the brain via vascular, peripheral nervous, lymphatic, cerebrospinal fluid pathways (45).

## The Important Link Between Proinflammatory Cytokines and Depressive Symptoms

Several studies have suggested that inflammation or immune dysregulation are implicated in the pathophysiology of depression (46–51). It is now well-established that both the innate and adaptive immune systems become dysregulated in depressed patients and that controlling inflammation might be of therapeutic benefit (52). Two meta-analyses showed reliably higher levels of inflammatory markers in depression, namely IL-1β, IL-6, C-reactive protein (CRP), and TNF-α (53, 54). Plasma CRP in depression was not only positively associated with plasma levels of inflammatory cytokines (e.g., IL-6, TNF-α, sTNFR2, and IL-1ra), but also correlated with the level of CRP in cerebrospinal fluid (55). Both Alexopoulos et al. and Galecki et al. reported continual interactions between changes in the peripheral immune response and central immune activation [e.g., macrophage accumulation and microglial activation; (56, 57)]. These central and peripheral immune changes lead to increased production of proinflammatory cytokines (58, 59), which in turn lead to abnormalities in brain circuits. To some extent, this permits the relationship between abnormalities in brain circuits and inflammatory states in depression to be inferred. Hao et al. demonstrated that psychiatric patients were significantly higher in their levels of worry, anger, impulsivity, and intense suicidal ideation than healthy controls during the peak of the COVID-19 epidemic (60). Based on the psychological impact of the COVID-19 pandemic on psychiatric patients, targeting the cholinergic anti-inflammatory pathway and modulating brain circuits using taVNS is a rational approach to treating COVID-19 and its associated cytokine storm. Controlling inflammation might provide an overall therapeutic benefit, regardless of whether it is secondary to early life trauma, a more acute stress response, microbiome alterations, a genetic diathesis, or a combination of these and other factors.

## Dysfunction of Cortico-Limbic-Striatal Circuits in Depression

Dysfunction of the cortico-limbic-striatal neural system, including cortical (anterior cingulate and prefrontal cortex) and limbic (amygdala, hippocampus, parahippocampal gyrus, cingulate gyrus, nucleus accumbens, and striatum) areas has been implicated in depression (61–63). Mayberg found dorsal and lateral cortical hypoactivity and ventral limbic hyperactivity in depression using positron emission tomography (64). Taylor and Liberzon also proposed a hypo-dorsal and -lateral cortical model of cognitive processes and a hyper-limbic model of emotional expression to account for the experience of depression (65). Using tasks requiring executive control and emotional information processing, Siegle et al. identified sustained increased amygdala activity in response to emotional information processing and decreased dorsal prefrontal cortex activity in response to executive cognitive tasks (66). Using a meta-analytic technique, Fitzgerald et al. identified two neural systems implicated in emotional regulation in depression, including reduced activity in dorsolateral prefrontal cortex and more dorsal regions of the anterior cingulate cortex (67). Furthermore, they found increased activity in medial prefrontal cortex and in subcortical regions related to emotional processing in the depressed state. All of these changes returned to normal after antidepressant treatment. Together, these studies imply that patients with depression may exhibit impairments in their cognitive control network, as evidenced by their inability to disengage from negative stimuli (68). In addition, they show impairments in their affective control network, as evidenced by the hyperactivity of their amygdala and hippocampus to negative stimuli and recall.

## The Stimulation Location of taVNS

Discrepancies in stimulation locations exist among studies that stimulated the auricular branch of the vagus nerve (69). The location is often dictated by the geometry of an electrode, with clip electrodes typically attached to the tragus or cymba concha (70–73). The outer auditory canal is also reported as a stimulation site, without further clarification for the electrode location (74–76). Based on Peuker and Filler's anatomical studies, the auricular branch of the vagus nerve innervates the tragus, concha, and cymba concha (77). However, it is difficult to select an optimal stimulation site for any particular disorder. The taVNS devices are relatively inexpensive, small, and mobile, which will be performed at patient's home after training (78).

## Stimulation Parameters for taVNS

As taVNS is a novel treatment, there is currently no consensus on the appropriate stimulation parameters for its therapeutic use. According to the latest published International Consensus on taVNS (79), the points stimulated by taVNS are located in the auricular concha region, which contains a rich distribution of vagus nerve branches. Stimulation parameters used in taVNS studies have included: (1) a 20-Hz continuous sinusoidal wave (wave width, 0.2 ms) (80, 81); a 10-Hz continuous sinusoidal wave (73); a 20–30 Hz continuous sinusoidal wave (82, 83); a 4/20 Hz dense wave (between 0.8 and 1.5 mA) (84); a 20 Hz dense wave (between 4 and 6 mA) (72, 85); 1.5 Hz unipolar rectangular waves (0–600 mA) (69); a 120 Hz pulse wave (12 mA) (86); a 25 Hz monophasic rectangular waves (87); and (2) a gradually increasing stimulation intensity, starting from zero up to the highest point that the patients could tolerate (typically between 4 and 6 mA) (12). In terms of the safety of taVNS, a systematic review by Redgrave et al. reported the side effects of taVNS as local skin irritation, headache, nasopharyngitis, and a number of potentially serious adverse events [e.g., palpitations; (88)]. Indeed, the vagus nerve projects to the parabrachial nucleus, which can regulate heart rate, with one study showing that taVNS can cause side effects on heart rate when specific stimulation parameters (pulse width, 500 μs; frequency, 25 Hz) are used (89). However, in most cases, side effects were not apparent or disappeared after follow up (86, 90, 91).

## Gender and Age-Dependent Differences for taVNS

VNS has greater effects in females in animal studies, probably because of the effect of estrogens on muscarinic acetylcholine receptors in the central nervous system (92). Similar effects would be expected in females human subjects due to both hormonal levels and the gender-dependent differences in the functions of the autonomic nervous system (93, 94). Age is associated with marked changes at the hormonal level, which in turn affect acetylcholine-mediated parasympathetic autonomic activity (95, 96). Fallgatter et al. reported that the vagus sensory-evoked potentials showed a trend toward reduction in the elderly, associated with age-related demyelination of neuronal structures or degenerative processes (97). In addition, sensitivity to electrical transcutaneous stimulation was found to be lower in the elderly (98).

## The Relationship Between Proinflammatory Cytokines and Brain Circuits in Depression

There is now accumulating evidence that different forms of proinflammatory cytokine-mediated communication between the immune system and brain circuits modulate the inflammatory pathway in the brain (99–101). Rodent and human neuroimaging studies combined with experimental inflammatory challenges have been successful in clarifying the sensitivity of the insula and striatum to changes in peripheral inflammation in depression (102). Of note, neuroinflammation is associated with structural and functional anomalies in depression (103). A negative correlation was found between CRP levels and the cortical thickness of the right medial prefrontal cortex (mPFC) in depression (104). In a recent resting-state functional magnetic resonance imaging (fMRI) study, CRP level was negatively correlated with amygdala–ventromedial prefrontal cortex (vmPFC) connectivity in depressed patients with high levels of inflammation and symptoms of anxiety (105). Haroon et al. demonstrated that plasma CRP levels are significantly associated with glutamate levels in the left basal ganglia using magnetic resonance spectroscopy (MRS) (106), and increased glutamate in the left basal ganglia in turn correlated with anhedonia and psychomotor slowing. Haroon et al. further pointed out that patients with high levels of both inflammation and basal ganglia glutamate showed decreased local homogeneity in vmPFC, and in dorsal and ventral striatal regions (107). In their study of medically stable patients with depression, Felger et al. reported that levels of CRP, as well as those of IL-6, IL-1beta, and IL-1ra, were negatively associated with connectivity between ventral striatum and vmPFC, and that this decreased connectivity in turn correlated with increased anhedonia (108). Moreover, the level of CRP was negatively correlated with connectivity between dorsal striatum, vmPFC, and presupplementary motor area. Decreased connectivity between dorsal striatum, vmPFC, and presupplementary motor area were further correlated with motor speed and psychomotor slowing. More recently developed methods, such as large-scale network-based analyses, were used by Yin et al. to show that the increased level of CRP is associated with reduced connectivity in ventral striatum, amygdala, orbitofrontal and insular cortices, and posterior cingulate cortex (109). Using surface-based morphometry, Kakeda et al. demonstrated that cortical thicknesses, such as those of the superior frontal and medial orbitofrontal cortex, showed a significant inverse correlation with the level of IL-6 (110). Using automated cortical parcellation within the mPFC including Brodmann areas (BA) 9, 10, 11, 24, 25, and 32, Meier et al. found an inverse relationship between plasma CRP level and the thickness of BA32, with recurrent MDD patients having a thinner cortex in BA32 (104). Using voxel-based morphometry, Chen et al. found that orbitofrontal cortex, lingual gyrus, inferior frontal cortex, middle frontal cortex, and planum polare were negatively correlated with levels of IL-6 (111). Moreover, Frodl et al. reported an inverse relationship of IL-6 concentration and hippocampal volume in MDD (112). Doolin et al. provided additional evidence to support a negative association between CRP levels and hippocampal subfield volumes (113). Importantly, the striatum, vmPFC, and presupplementary motor area are part of the classical reward and motor circuitry that receives neurotransmitters such as glutamate, in addition to dopaminergic innervation (114–116). Furthermore, Nusslock et al. found that higher levels of inflammatory biomarkers (e.g., CRP, IL-6, IL-10, and TNF-α) were associated with lower connectivities within both the emotional network and the central executive network in urban African American youths, suggesting that inflammation or neuroimmunology may be involved in the pathogenesis of emotional and physical health problems (117). More importantly, Cosgrove et al. reported that higher levels of CRP were related to greater coupling of orbitofrontal cortical and anterior insular activity with increased appetite in depressed patients (118). Together, these studies imply that systemic low-grade inflammation is associated with the coupling of activity in striatum with that in reward- and interoceptive-related neural circuitry, and provide evidence for physiological subtypes within depression.

## Effects of taVNS on the Limbic-Cortico-Striatal-Thalamo-Cortical Circuits to Address the Depressive Symptoms of Covid-19

Macrophages, proinflammatory cytokines (such as interleukin (IL)-1β, IL-6, and tumor necrosis factor (TNF)-α) and chemokines released by respiratory epithelial and dendritic cells, are all known to play a role in the pathogenesis of critical patients with COVID-19 (119). Consequently, Bonaz et al. hypothesized that targeting the cholinergic anti-inflammatory pathway by vagus nerve stimulation could be a useful therapeutic option for patients with COVID-19. In support of this hypothesis, Staats et al. recently reported two patients with respiratory symptoms that were similar to those associated with COVID-19 who showed marked clinical benefit following the application of transcutaneous cervical vagus nerve stimulation (19). Research has also shown that the levels of proinflammatory cytokines, including IL-6, IL-10, IL-12, IL-13, and TNF-α, are elevated in MDD when compared to those of healthy controls (120). However, there is still a clear shortage of evidence supporting the neuroimaging findings of taVNS in the treatment of depressive symptoms in patients with COVID-19. Our previous review has validated taVNS may inhibit both peripheral and central inflammation and modulate multiple neural systems (121). Studies have demonstrated that taVNS increases connectivity of the nucleus accumbens (NAc) with bilateral mPFC/rostral anterior cingulate cortex (rACC); NAc with insula, occipital gyrus, and lingual/fusiform gyrus; amygdala with dorsolateral prefrontal cortex; and the default mode network (DMN) with precuneus and orbital prefrontal cortex. In addition, studies have reported decreased connectivity of medial hypothalamus (MH) with rACC, and DMN with anterior insula and parahippocampus (72, 85, 122, 123). Therefore, we argued that it was advantageous for treating the inflammatory processes associated with COVID-19 and modulate brain activity in the NAc, hypothalamus, DMN, amygdala, and rACC via the auricular branch of the vagus nerve (78). Further, it has been suggested that taVNS can attenuate inflammation by targeting the HPA axis (16).

Finally, since the beginning of the COVID-19 pandemic, various manifold neuroimaging features have been described for patients with COVID-19 and a range of interesting and helpful findings have been described across the globe (124). For example, Jain et al. found that acute stroke was the most common finding on neuroimaging; 92.5% of patients with positive neuroimaging studies also showed evidence of acute stroke on neuroimaging. Acute stroke is therefore a strong prognostic marker for a poor outcome (125). In another study, Mao et al. reported that 36.4% of patients had headache, dizziness, impaired consciousness, acute cerebrovascular disease, ataxia, and seizures, and that 8.9% of patients experienced specific manifestations in their senses, including taste, smell, vision impairment, and nerve pain (126). Furthermore, Brouwer et al. reported that acute cerebrovascular events were also detected in ~3% of patients and that 6% of patients with severe manifestations had cerebrovascular events (127). Similarly, Tsai et al. reported a wide range of neurological manifestations, including olfactory taste disorders, headache, acute cerebral vascular disease, dizziness, altered mental status, seizure, encephalitis, neuralgia, ataxia, Guillain-Barre syndrome, Miller Fisher syndrome, intracerebral hemorrhage, polyneuritis, and dystonic posture (128). In addition, Al-Olama et al. reported that COVID-19 infection can cause meningoencephalitis in right frontal intracerebral hematomas, subarachnoid hemorrhage, and in frontal and temporal lobe thin subdural hematomas (129). Therefore, obtaining detailed neurological examinations and neuroimaging for the early and accurate diagnosis of these often fatal neurological complications could significantly improve our understanding of COVID-19 and its neurological manifestations.

## Effects of taVNS on the Cholinergic Anti-Inflammatory Pathway and Hpa Axis

**Figure 2:**
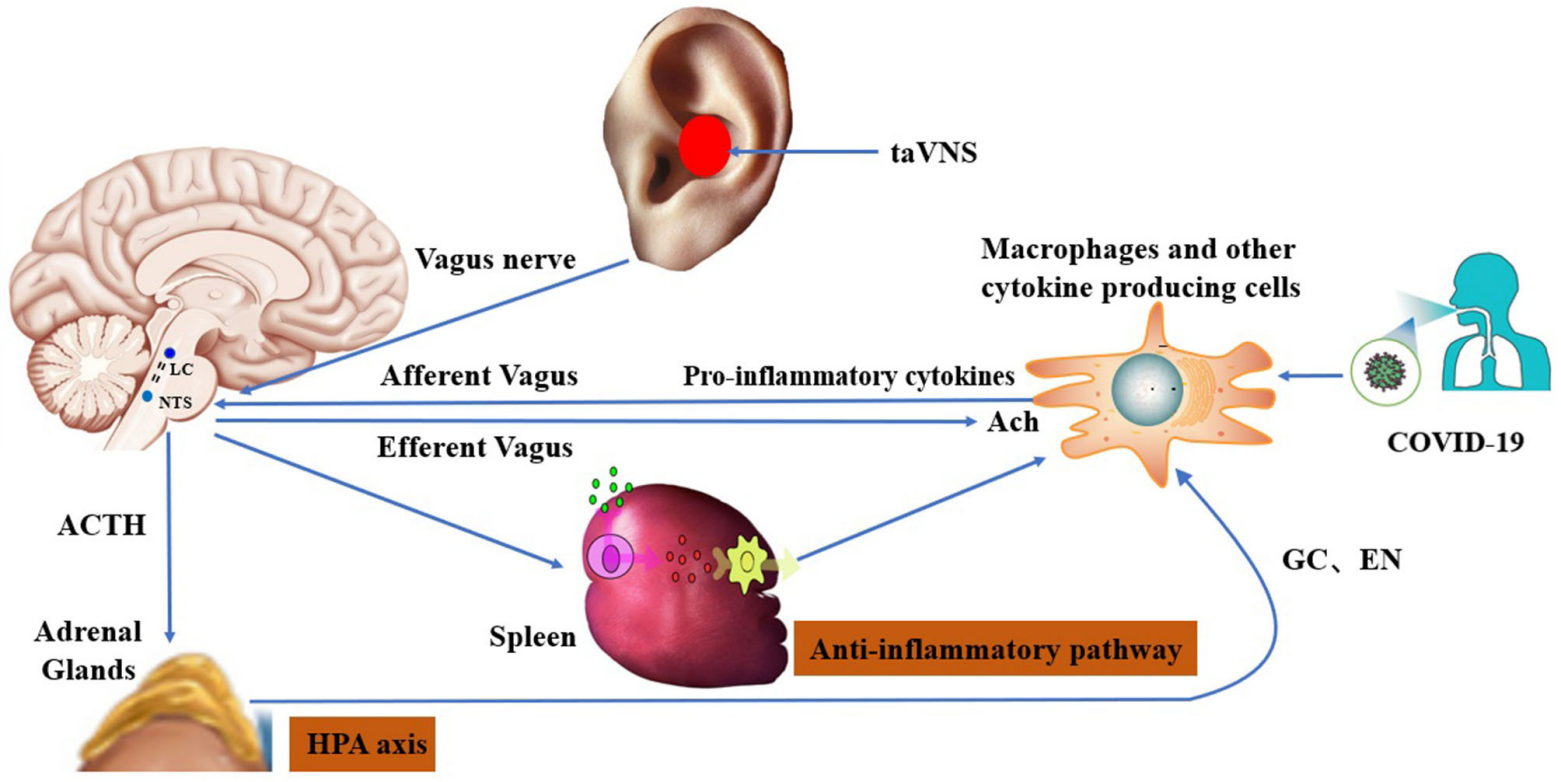
Hypothesized mechanisms of transcutaneous auricular vagus nerve stimulation in the treatment of post-COVID-19 sequelae: 1) improvement in immune function via the cholinergic anti-inflammatory pathway; and 2) modulation of brain circuits via the HPA axis [according to Bonaz and Sinniger, 2016 (134)].

The cholinergic anti-inflammatory pathway via the vagus nerve has been proposed to be a key mediator of cross-communication between the peripheral immune system and the brain (130). Indeed, an increase of TNF-α in the liver and blood induced by an extreme immune reaction or cytokine storm was successfully dampened by stimulation of the vagus nerve, inducing an anti-inflammatory effect involving the release of acetylcholine (ACh) (131). Promisingly, Staats et al. reported clinically meaningful benefits of VNS in two COVID-19 patients with severe acute respiratory syndrome (19). The vagus nerve has a dual anti-inflammatory role, with 80% of the afferents targeting the cholinergic anti-inflammatory pathway and 20% of efferent fibers targeting the HPA axis (132). The efferent fibers of the vagus nerve activate the HPA axis, causing glucocorticoid release from the adrenal glands (133). Efferent fibers also run through the neck, connecting the brainstem to many organs, including the spleen, where they inhibit the release of TNF-α (16). Targeting the vagus nerve non-invasively may open up novel adjuvant approaches to treating COVID-19 patients. The various mechanisms by which taVNS may treat inflammation and related organ dysfunction in COVID-19 are illustrated in Figure 2.

## Traditional Chinese Medicine (TCM) on Covid-19

TCM has a history of more than 2,000 years in the prevention and treatment of epidemics and plagues and the national health commission of China has recommended some patent Chinese medicine, such as Jinhua Qinggan granules, Lianhua Qingwen capsules, Xuebijing injections, a Qingfei Paidu decoction, a Huashi Baidu decoction, and a Xuanfei Baidu decoction (135). Patients with COVID-19 who took Jinhua Qinggan granules recovered faster than those who did not take the granules (136). Therapeutic efficacy was significantly higher in patients with COVID-19 taking Lianhua Qingwen capsules and Arbidol (umifenovir) than that in those taking Arbidol alone; moreover, the conversion rate to severe disease in patients taking these capsules was significantly lower than that in patients taking Arbidol alone (137). Furthermore, chest computed tomography images of patients with COVID-19 showed improvement after 6 days of treatment with Qingfei Paidu decoction (138). In addition, other therapies such as acupuncture might also play a beneficial role in treating breathlessness after COVID-19 (4). Thus, TCM could play an important role in fighting COVID-19 in China.

## Conclusions

This review has provided a comprehensive evaluation of targets for taVNS that can be used to treat inflammation and related organ dysfunction in COVID-19. It is clear that COVID-19 involves interrelationships between proinflammatory cytokines and brain circuits. The research findings detailed here suggest that taVNS could be used as an adjuvant therapy for depressive symptoms during the COVID-19 pandemic. We present a rationale for targeting the anti-inflammatory process and modulating brain circuits to treat COVID-19 and its associated cytokine storm. The evidence we present suggests that in theory, in response to the respiratory symptoms and immune system damage caused by COVID-19, taVNS can be used to improve immune function and may be an important treatment for depressive symptoms on post-COVID-19 sequelae. We describe the multi-level mechanisms linking taVNS and regulation of systemic anti-inflammatory responses and prevention of neuroinflammation present so as to treat depressive symptoms during the COVID-19 pandemic. When pro-inflammatory cytokines are present due to an infection, taVNS can activate afferent vagal neurons through impacting the immune response (139, 140) and also efferent vagal neurons can release acetylcholine through the cholinergic anti-inflammatory pathway and HPA axis (132, 141). Then, we summarize how applying taVNS and targeting cognitive and mental distress through influencing the connectivity of neural networks (121). taVNS has been shown to be associated with improved the default mode network functioning, which has been implicated in cognitive as well as emotional functioning (72, 142). Further studies are needed to understand the relationship between the immune system and the brain, as well as the role of taVNS.

## Author Contributions

This paper was primarily written by C-HL, PS, and Z-PG. Figures were produced by C-HL, Z-PG, Z-QZ, DL, and M-HY. All authors read and approved the final manuscript.

## Funding

This work was supported by grants from the Municipal Natural Science Foundation of Beijing of China (Grant No. 7212200) and National Natural Science Foundation of China (Grant Nos. 81871507 and 81471389).

## Conflict of Interest

The authors declare that the research was conducted in the absence of any commercial or financial relationships that could be construed as a potential conflict of interest.

## Publisher's Note

All claims expressed in this article are solely those of the authors and do not necessarily represent those of their affiliated organizations, or those of the publisher, the editors and the reviewers. Any product that may be evaluated in this article, or claim that may be made by its manufacturer, is not guaranteed or endorsed by the publisher.

## Abbreviations

ACh: acetylcholine
AMY: amygdala
ARDS: acute respiratory distress syndrome
BA: Brodmann areas
CCL-2: C-C motif chemokine ligand
COVID-19: coronavirus disease 2019
CRP: C-reactive protein
EN: epinephrine
fMRI: functional magnetic resonance imaging
GC: glucocorticoid
HIP: hippocampus
HPA: hypothalamic pituitary adrenal
IL: interleukin
IL-1ra: interleukin-1 receptor antagonist
LC: locus coeruleus
MDD: major depressive disorder
MH: medial hypothalamus
mPFC: medial prefrontal cortex
MRS: magnetic resonance spectroscopy
NAc: nucleus accumbens
NE: noradrenaline
NTS: nucleus tractus solitarius
PFC: prefrontal cortex
PTSD: post-traumatic stress disorder
rACC: rostral anterior cingulate cortex
taVNS: transcutaneous auricular vagus nerve stimulation
TCM: Traditional Chinese Medicine
TNF: tumor necrosis factor
vmPFC: ventromedial prefrontal cortex
VNS: vagus nerve stimulation.
